# Lower Limb Degloving Trauma Reconstruction: A Case Report

**DOI:** 10.7759/cureus.48904

**Published:** 2023-11-16

**Authors:** Alan Armando Sosa-Vazquez, Yeudiel Suro Santos, Jorge Alejandro Serrato-Ruiz, Jaime Aaron Herrera-Valenzuela, Agustin Arturo Becerril-Pazara, Victor Carlos Hayakawa-Davila

**Affiliations:** 1 Plastic and Reconstructive Surgery, Instituto Mexicano Del Seguro Social, Unidad Medica de Alta Especialidad 71, Universidad Autonoma de Coahuila, Torreon, MEX; 2 General Surgery, Instituto Mexicano del Seguro Social, Hospital General de Zona No. 33, Monterrey, MEX; 3 Plastic and Reconstructive Surgery, Instituto Mexicano del Seguro Social, Unidad Medica de Alta Especialidad 71, Universidad Autonoma de Coahuila, Torreon, MEX

**Keywords:** acellular dermis, negative-pressure wound therapy, leg injuries, skin transplantation, degloving injuries

## Abstract

Degloving injuries are caused by trauma with shear mechanisms, usually secondary to being run over in traffic accidents. It is characterized by the avulsion of skin and subcutaneous tissue, generating coverage deficit, and in severe cases, loss of the affected limb. Herein, we discuss a case of a six-year-old male run over by a truck, receiving a direct impact on the left lower extremity with a rubber tire. Debridement and surgical cleaning were performed and a negative pressure system was placed. Subsequently, dermal matrix and partial-thickness skin grafts were placed as coverage after the creation of a viable grafting bed.

## Introduction

Degloving injuries result from traumatic shear mechanisms and usually affect the lower extremities; they are often caused by traffic accidents or sports-related incidents [1]. These injuries involve the avulsion of skin and subcutaneous tissue, which can lead to compromised blood supply, tissue necrosis, coverage deficits, and, in severe cases, limb loss [2]. Prompt surgical intervention aims to preserve the limb, ensure quality coverage, and minimize potential functional complications.

This case involves a pediatric patient who suffered an extensive degloving injury in the left lower extremity due to a truck accident. The reconstruction process required the use of an acellular dermal matrix, partial-thickness skin grafts, and negative pressure wound therapy.

## Case presentation

**Figure 1:**
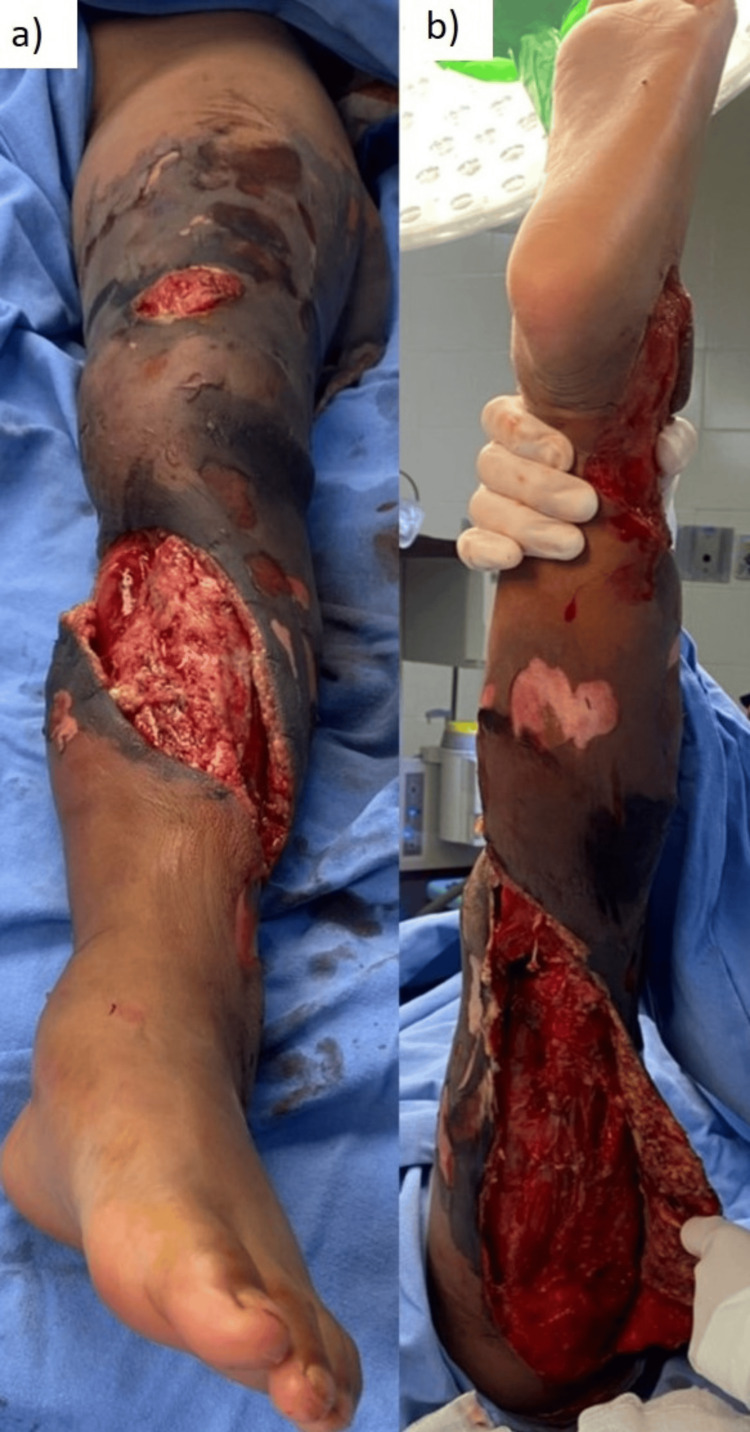
Left lower extremity with degloving injury. (a) Anterior view; (b) posterior view

A six-year-old male with no comorbidities was involved in a truck accident, receiving a direct impact to his left lower extremity with a rubber tire. He was transported to a referral hospital in Torreón, Mexico. Upon examination, avulsed dermal and fatty tissue flaps were observed, showing signs of ischemia, with areas of exposed muscles and tendons in the thigh, knee, and leg regions. The injury extended circumferentially from the trochanter to the ankle, with no apparent damage to the neurovascular bundle (Figure 1).

The patient had intact pulses in the left lower extremity, preserved sensation and strength, no active bleeding, an unaffected left hip, and moderate foot swelling. The contralateral extremity had no abnormalities, and radiological studies revealed no fractures. Laboratory results showed slight leukocytosis, with hemoglobin levels within the normal range.

**Figure 2:**
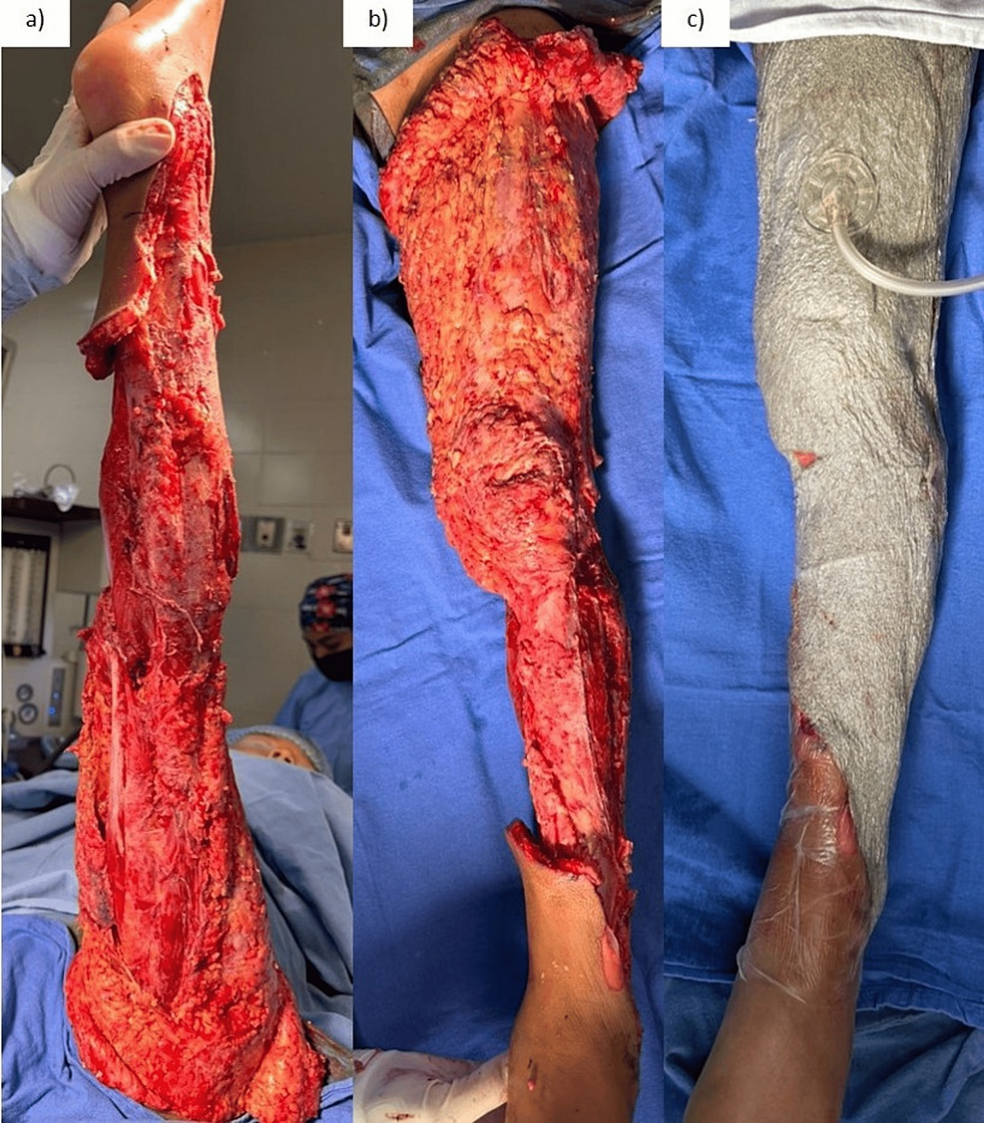
First surgical intervention which consisted of debridement and placement of negative pressure therapy. (a-b) debridement of non-viable tissue; (c) placement of negative pressure therapy

Urgent surgical debridement and cleansing were performed, resulting in a clean wound bed across the thigh, proximal third, and mid-leg (Figure 2).

Due to the extensive coverage deficit, a negative pressure system using "silver foam" and "white foam" at a continuous pressure of 125 mmHg was applied. Surgical cleanings and changes of the negative pressure system were performed on Days 4 and 9 after the initial surgery until a viable wound bed with adequate granulation tissue and no signs of infection were achieved.

**Figure 3:**
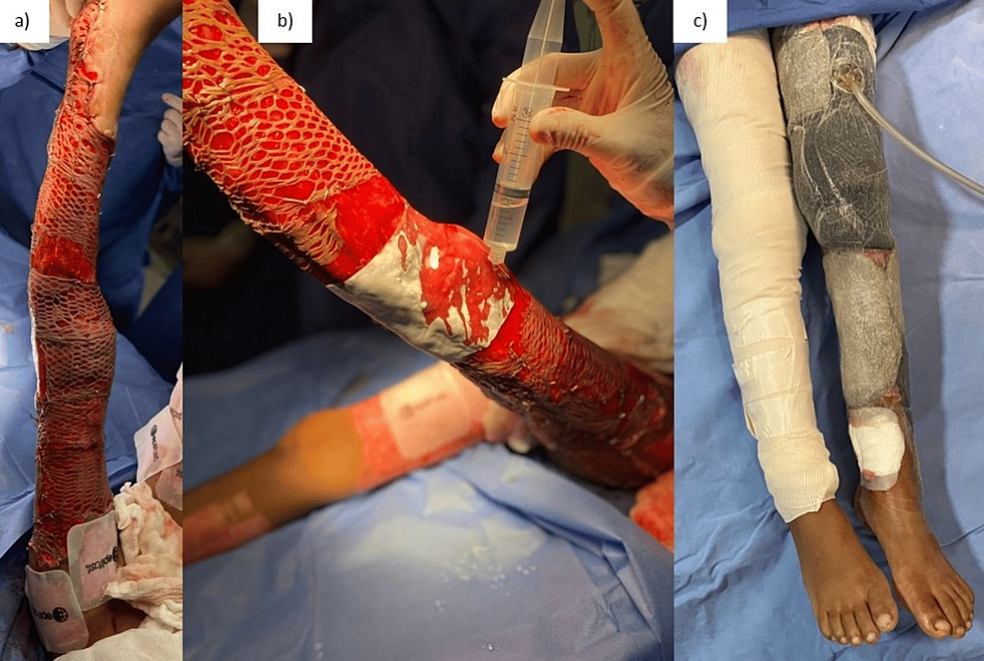
Skin grafts and acellular dermal matrix placement on Day 14 after first surgery (a) Placement of partial thickness skin grafts; (b) acellular dermal matrix was placed at the femorotibial joint; (c) skin graft coverage with negative pressure therapy

On Day 14, an acellular dermal matrix was placed at the femorotibial joint (knee), followed by intermediate partial-thickness skin grafts (0.30 mm) on the matrix and areas of the wound. These grafts were secured with metal staples and covered with in vitro-cultured human epidermis allograft patches (Epifast®). Negative pressure therapy in dynamic mode at 100 mmHg was initiated (Figure 3).

**Figure 4:**
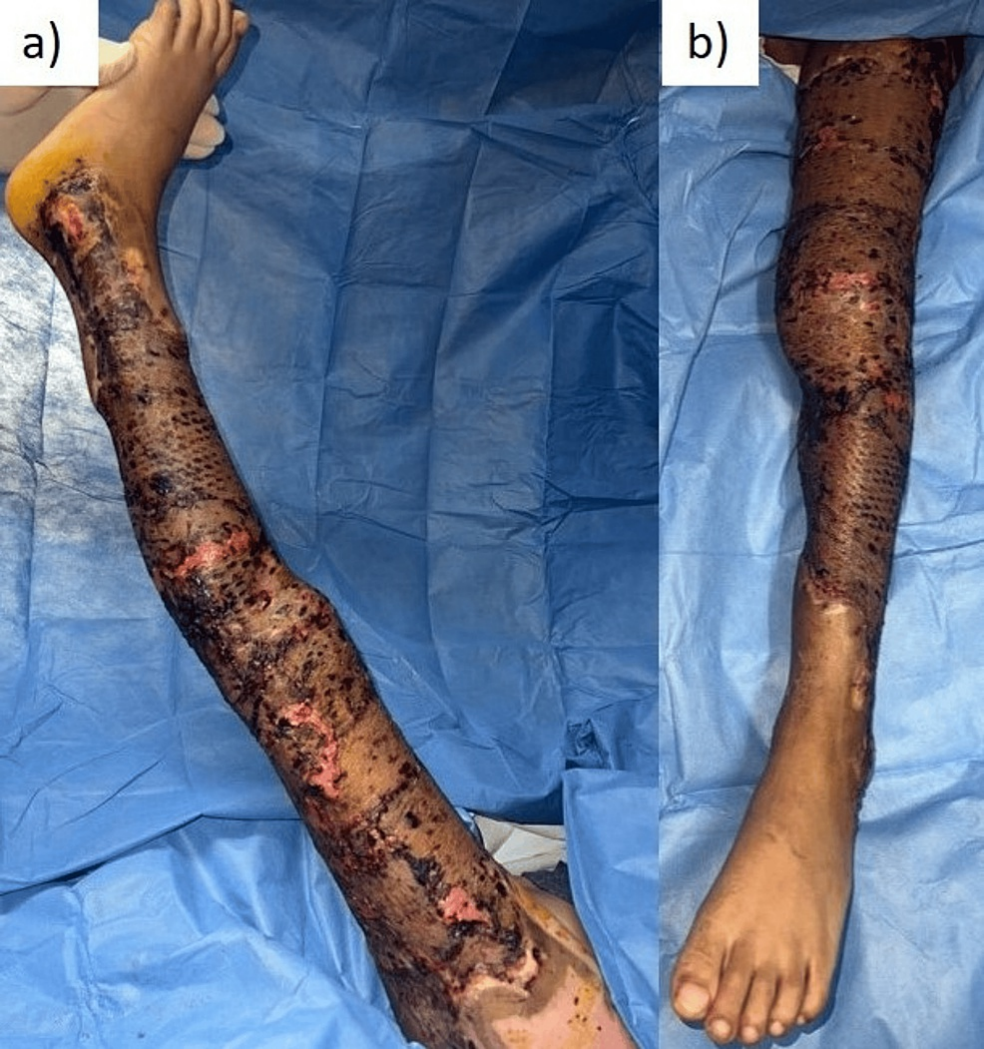
Day 5 after skin graft surgery (Day 19 after the first surgery). (a) Left leg lateral view; (b) left leg anterior view

On Day 19, the negative pressure system was removed, revealing fully integrated skin grafts with no exposure of muscle or tendons (Figure 4).

**Figure 5:**
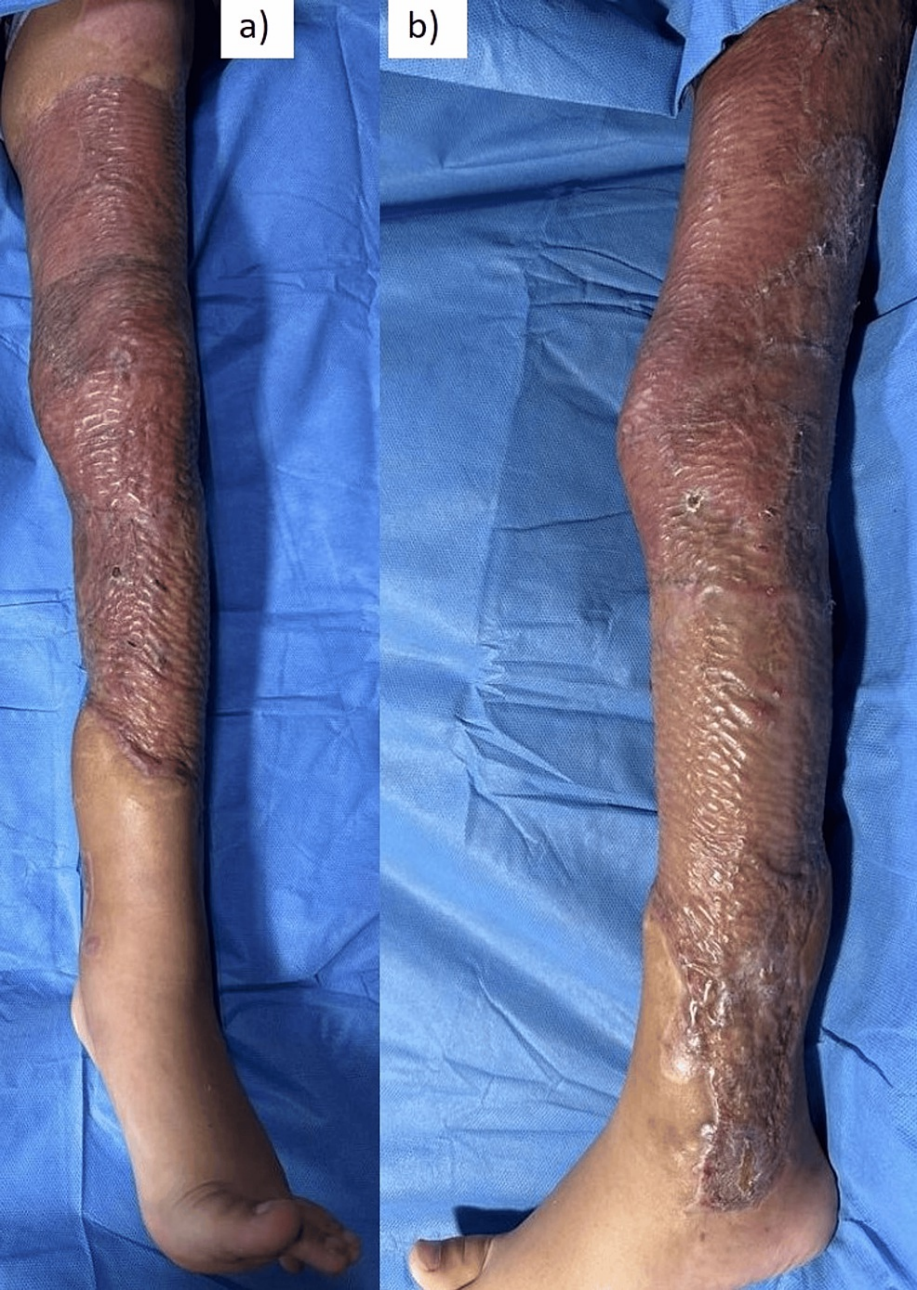
Follow-up three months after leg trauma. (a) Anterior view; (b) lateral view

The patient continued to be monitored by the plastic and reconstructive surgery department, along with the department of rehabilitation, resulting in an excellent outcome three months after the definitive coverage placement (Figure 5).

The patient regained satisfactory knee joint mobility, with a flexion capacity of up to 70 degrees, an ankle range of motion of 50 degrees, and a toe flexion capacity of 40 degrees, enabling normal ambulation and jumping.

## Discussion

Degloving injuries are associated with high morbidity and mortality when treated inadequately [2]. While they can occur in various body areas, the lower extremities are the most common site [3]. The treatment of extensive lower extremity degloving injuries aims to preserve the limb, restore function, enhance aesthetic appearance, and provide appropriate coverage [4,5]. Degloving injuries can be classified into three categories: pure degloving (skin only), degloving injuries involving deep soft tissues (muscle and deep fascia), or degloving injuries associated with fractures [2]. The initial assessment includes evaluating the vascular and nervous viability of soft tissues, lesion location, the presence of infection, and fractures [6].

Initial steps involve the debridement of non-viable and contaminated tissue. Once the infection is controlled and an adequate wound bed is established, definitive coverage can be applied. Reconstruction should be performed as soon as possible, ideally within the first two weeks, to minimize morbidity [4]. However, many cases require multiple surgical interventions to prepare a viable grafting bed.

In cases where skin flaps are viable, they can be reattached if they are found to be in adequate condition. In this case, the avulsed skin flap exhibited ischemic changes, hematomas, and areas of necrosis, necessitating extensive resection of non-viable tissue.

Multiple options exist for covering extensive defects, such as allografts, flaps, skin grafts, skin substitutes, or biological dressings [6]. The use of negative pressure wound therapy, also known as vacuum-assisted closure or VAC as well as acellular dermal matrix and grafts allows for wound coverage and faster recovery with low morbidity [7,8]. The acellular dermal matrix acts as a scaffold for capillary and cellular proliferation, creating a dermal equivalent that facilitates wound reepithelialization and neovascularization. It is mainly used in poorly perfused wound beds, such as tendons, cartilage, and bone, before placing a graft [7,9]. This approach provides higher-quality coverage compared to isolated partial-thickness grafts. We used meshed grafts instead of sheet grafts because the coverage area was too large.

Negative pressure therapy accelerates wound granulation, reduces edema and wound surface area, and decreases infection rates. It promotes wound bed perfusion and graft integration [4,8]. Pressure levels ranging from 75 mmHg to 125 mmHg have been used in degloving injury treatment [10]. 

## Conclusions

Degloving injuries in the lower extremities require complex and individualized reconstruction to avoid functional complications, aesthetic issues, or limb loss. The use of an acellular dermal matrix provides high-quality coverage in joint regions, preventing contracture-related stiffness and ulceration that can cause exposure of joint structures. In this case, a combination of negative pressure therapy, acellular dermal matrix in the joint region, and skin graft application optimized the reconstruction timeline, reducing hospital stay and morbidity. The patient achieved excellent aesthetic and functional results three months after definitive coverage placement.

## References

[1] Kushare I, Ghanta RB, Wunderlich NA (2021). Morel-Lavallée lesions (internal degloving injuries) of the lower extremity in the pediatric and adolescent population. Phys Sportsmed.

[2] Yan H, Gao W, Li Z, Wang C, Liu S, Zhang F, Fan C (2013). The management of degloving injury of lower extremities: technical refinement and classification. J Trauma Acute Care Surg.

[3] Latifi R, El-Hennawy H, El-Menyar A, Peralta R, Asim M, Consunji R, Al-Thani H (2014). The therapeutic challenges of degloving soft-tissue injuries. J Emerg Trauma Shock.

[4] Zeiderman MR, Pu LL (2021). Contemporary approach to soft-tissue reconstruction of the lower extremity after trauma. Burns Trauma.

[5] Baş S, Öztürk S, Akbulut HA, Öner Ç (2022). Lower extremity reconstruction: 5 years of clinical experience. zmir Tıp Fak Derg.

[6] Jordan DJ, Malahias M, Hindocha S, Juma A (2014). Flap decisions and options in soft tissue coverage of the lower limb. Open Orthop J.

[7] Shakir S, Messa CA 4th, Broach RB (2020). Indications and limitations of bilayer wound matrix-based lower extremity reconstruction: a multidisciplinary case-control study of 191 wounds. Plast Reconstr Surg.

[8] Hallock GG (2013). Evidence-based medicine: lower extremity acute trauma. Plast Reconstr Surg.

[9] Iorio ML, Shuck J, Attinger CE (2012). Wound healing in the upper and lower extremities: a systematic review on the use of acellular dermal matrices. Plast Reconstr Surg.

[10] Barendse-Hofmann MG, van Doorn L, Steenvoorde P (2009). Circumferential application of VAC on a large degloving injury on the lower extremity. J Wound Care.

